# Puerperal Phlebitis—Purulent Infection—With Remarks

**Published:** 1859-05

**Authors:** J. J. Thweatt

**Affiliations:** Petersburg, Va.


### SELECTIONS.

*Puerperal Phlebitis—Purulent Infection—With Remarks.* By J. J. Thweatt, M. D., Petersburg, Va.

Case I.—May 9th, 1857.—We were requested to visit a negro woman, æt. 35, who had been confined a few days previous. Her owner gave the following history of her case: She had enjoyed good health during pregnancy, and, her labor had been easy, and unattended by any accident. On the fourth day after accouchement she got up and attended to dressing her child. In a few hours afterwards she complained of being cold; thinks she had a distinct chill; the chilliness terminated in a clammy sweat; she complained of lassitude, was restless and excited; now she said her leg and thigh hurt her, which soon began to swell, and the leg was painful when roughly handled. At night she had fever, and slept but little; a dose of oil had been given her, which was the only medicine she had taken up to the time I saw her. Her condition, when we saw her, was well calculated to excite serious apprehensions. The expression of countenance was haggard and anxious; skin bathed in a cold, clammy perspiration; pulse quick, but compressible; tongue pointed, moist; free from fever; complained of pain in the leg, which, on examination, was found much swollen, and acutely sensible to the touch; the veins prominent and hard; the abdomen was slightly tympanitic, but pressure caused no pain or complaint. The uterus was evidently larger than it should be at this period of her confinement, but the entire uterine region was insensible to pressure; the respiration was hurried; pulsations of the heart rapid, but no abnormal sound; there was an entire cessation of the lochial discharge. In addition to the pain in the leg, the patient complained of pains in the elbows, wrists and fingers; the tendons of the fingers were partially contracted.

She was directed to take a dose of calomel, quinine and opium; to apply hot fomentations to the limb; soup.

On our next visit we found all the symptoms aggravated; the patient had passed a sleepless night; was delirious during a great part of it; her countenance wore a more haggard and anxious expression; pulse very rapid; respiration very quick, accompanied with sighing and groaning; complained more of the pains in the leg, arms and wrists; the latter were swollen and tender; her leg was more swollen and tender; and the surface covered with phlyctæna; the internal saphenous vein felt like a cord; the cellular tissue was œdematous; the abdomen remained still insensible to pressure. Ordered brandy and wine every two hours; beef tea, &c. The patient died next morning.

Case II.—May 14th, 1857.—We were summoned in haste to see Mrs. D. This lady was the mother of five or six children; her constitution excellent, and her general health had been good; her labors had always been free from difficulty or accident; she had given birth to a healthy child a few days before we saw her. On the fourth day, feeling very well, she got up and attended to some domestic affairs; the next day she was seized with a chill, with pains in the back and extremities. When we saw her, she was very much excited, and expressed great fear with regard to her condition; her feelings she said, were indescribable; to use her own emphatic language, "I do feel so curious, I do not know where I am sick, but I do know that I am very sick." She was very restless, throwing herself from one side of the bed to the other, uttering frequent exclamations of alarm; skin hot; pulse quick and irritable; tongue clean, disposed to be dry; complains of headache and lassitude; respiration hurried, heart beating rapidly. No pain was manifested on a minute exploration of the abdominal and uterine regions, but there was an evident increase of volume of the womb. We prescribed a febrifuge mixture. Saw the patient in the afternoon. She was more comfortable; skin moist; pulse still very quick; no thirst; complains of weariness. Directed calomel, quinine and opium.

15th.—The patient passed a restless night; complains now of pains in the arms and lower extremities; extremely anxious about her condition; countenance pale and contracted. Ordered quinine, five grains every three hours.

Visited her again in the afternoon, and found no improvement.—She was suffering more from pains in the limbs, which, on examination, were swollen and tender. Directed mint julep and a fnll anodyne at bed time; the limbs to be rubbed with chloroform liniment, and hot fomentations to be applied.

16th.—The anodyne had had very little effect; she was delirious during a great portion of the night; she was in a cold, clammy sweat; pulse could scarcely be counted; great jactitation; respiration more frequent; the inferior extremities, especially the thighs, were very tender to the touch; the saphenous vein was hard and tender, and there was a red streak all along its track; the lymphatics were also involved; the hand and wrist were swollen, and the slightest pressure produced intense pain. Prescription—julep, quinine and beef tea; the limbs to be covered with a poultice of stramonium leaves. She died early on the 17th.

The diagnosis in the above cases was puerperal suppurative phlebitis. The diagnosis had to be made out from the symptoms alone, as permission could not be obtained to make autopsies. This is to be regretted; for phlebitis in any form is a rare disease with us, and the symptoms are not always so obviously characteristic as to remove all chances for errors. If, however, a rigid analysis be made of the symptoms, we think there will be little doubt of the correctness of the diagnosis.

Both patients were seized, without any premonition, with a distinct chill, terminating in a few hours in viscid perspirations. The pulse was quick and irritable in both cases. It was at no period full, corded, or wiry, as in peritonitis or metro-peritonitis. The abdominal symptoms were null. The uterus, with the exception of a slight enlargement, was free from any ostensible disease. If there was any hesitancy with regard to the nature of the disease on the first and second days, all doubt and hesitancy were removed on the third day, by the appearance of indubitable signs of venous inflammation. The veins were hard, prominent and tender, and their course distinctly marked out by a red line. The cellular tissue was also engorged and œdematous. We had also unmistakable symptoms of purulent deposites in the articulations of the elbows, wrists, and probably the lungs and liver. There were also delirium, jactitation, insomnia, with all the physiognomical characteristics of great suffering.

In the case of the negro woman, we had phlegmasia dolens, a disease which is, by general consent of the best pathologists, acknowledged to consist in inflammation of the veins especially of the uterus.

Inflammation of the veins is a subject of great importance, and for the last ten or fifteen years has earnestly engaged the attention of some of the brightest intellects of the profession. If we are to rely upon the assertion of M. Velpeau, who is high authority on all questions connected with medical bibliography, it is a disease which was little known or studied by the fathers of our science. For a comprehensive knowledge of its pathology, we are indebted to the labors of Hunter, Ribes, Legallois, Velpeau, et cetera; and for its special connection with puerperal diseases, to Doctors Dance and Robert Lee.

This paper is designed to offer some reflections on phlebitis as a dis tinct puerperal disease, and not on inflammation of the veins in general. We have already directed the attention of the profession, through the pages of this journal, to a specific form of puerperal disease, viz.: puerperal rheumatism; and in that paper we stated, as our conviction, that there were various forms of puerperal diseases, which, although often blended, were separate in their pathology and therapeutics.—Among them were enumerated puerperal fever, puerperal peritonitis, puerperal phlebitis, &c. We little thought, when writing that article, that it would be our unfortunate task so soon to record two cases of that formidable disease, puerperal phlebitis.

This, like ordinary phlebitis, presents two distinct varieties—an adhesive and suppurative variety. In the first we have a local inflammation of the veins, which results in an obliteration of their cavities by the process of adhesion or formation of clots. The symptoms which mark this variety of the disease, are so plain and palpable and so well treated of by authorities, that it would be tedious to dilate upon them here. We will therefore simply remark, in reference to this variety, that it is generally tractable in its nature and under the control of a judicious application of remedies. But of puerperal suppurative phlebitis, with its almost inevitable result, purulent infection, we have to contend with the most rebellious, the most fatal disease in the whole catalogue.

The frequency of sudden deaths after surgical operations, and sometimes from extensive suppurating wounds, and also from the necroscopic phenomena which were almost constantly found, viz.: purulent deposites in the lungs, liver and articulations, led M. Velpeau, as far back as 1818, during his service at Tours, to enter into an investigation of their etiology. The results of investigation were published in 1821 and 1823. He announced the following propositions:

1. That the sudden deaths after operations were owing to the introduction of pus into the circulation.
2. That the pus was deposited in the vascular organs and tissues.
3. That the blood was impoisoned by the pus.

The announcement of these opinions, which were subsequently amply verified by the researches of Marechal, Reyneaux, Legallois, Rochoux, Sanson and Cruveilhier in France, Wagner in Germany, and Dr. Arnott in England, directed investigation into the causes of sudden deaths in lying-in women; and also into a more thorough study of the nature of puerperal fever. For at that time, and we may say with truth, at this period, all sudden deaths after parturition or during confinement, were ascribed to puerperal fever. Drs. Dance and Lee were the first to promulgate the fact, that inflammation of the veins of the uterus was frequently the cause of death in lying-in women, and thus introduced into the nosology of puerperal diseases, puerperal phlebitis.

The great author of physiological medicines deserves the gratitude of the profession of the present day for his useful efforts in establishing morbid phenomena upon a local basis. The localization of diseased actions was a theme on which he expended the force of his gigantic mind. Whatever the errors of that great man may be, and we freely admit that their name "is legion," no one can deny the value of his favorite aphorism, "Fix your disease." We therefore maintain that puerperal suppurative phlebitis is a distinct form of puerperal disease; that it may present itself in an isolated state, uncomplicated with any other disease; but frequently it is united with inflammation of the peritoneum, uterus, and the adjacent organs. The symptoms show conclusively that the blood is contaminated, and that this depraved condition of the blood is owing to the introduction of purulent matter. There are some curious points in the pathology of purulent infection (we use this term instead of purulent absorption, as we shall presently show that the latter is an impossibility), to which attention is now directed. All pathologists are of one accord relative to the contamination of the blood by the pus, but there is far from an unanimity of sentiment in relation to the mode by which the pus is carried into the circulation. Two prominent theories exist upon this subject, each of which is supported and defended by physicians of high talents and profound erudition.

It is maintained by Marechal, Legallois and others, that there is only one mode, or rather that this mode is sufficient to explain all the phenomena of purulent infection, viz.: absorption of pus by the veins, and from wounded surfaces. On the other side, Blandin, Arnott and Berard contend that inflammation of the veins is an essential prerequisite. Velpeau and Sanson are of the opinion that purulent matter is carried into the circulation by both modes; also by aspiration and by the lymphatics. A logical examination of these theories and opinions cannot fail to bring the unprepossessed mind to the conclusion that the secretion of pus in the cavities of the veins, the result of previous inflammation, is the most plausible and philosophic one.

M. Velpeau, whose opinions should always command attention and respect, relies on dissections to show that inflammation of the veins is not always necessary ; but to prove this, all the veins in the body should be dissected—an amount of labor which it would be difficult to find one to undertake. The theory of absorption must yield to the following facts : The function of absorption either by the veins or lymphatics, is characterized by great rapidity of action. All experiments demonstrate that substances, when presented to absorbing surfaces, are almost instantly absorbed. Again—If it were true that purulent infection was owing to the absorption of pus by the absorbing vessels, it would be a very common disease ; indeed, few would escape. On the contrary, it is not a very common disease, either in a surgical or obstetrical point of view. With respect to the absorption of pus globules, we answer, that this is an impossibility, for the experiments of Donné and Maudl prove, that the pus globules cannot be absorbed by the veins, for the simple reason, that their volume presents an insuperable barrier. We think the remark of M. Berard on this point not too strong, when he says, "les dimensions du globule du pus sont telles qu'il, faudrait etre stupide pour supposer que ces globules pruissent pennetrer au travers des parois vasculaires."

Allusion has already been made to the frequent necroscopic results of purulent infection, viz.: metastatic abscesses in the lungs, liver, brain, cellular and synovial membranes. The origin of these purulent deposits is involved in great mystery. Various opinions have been put forward in explanation of their mode of formation. Velpeau believes that pus globules may be absorbed, and deposited in an unaltered condition in the organs and tissues. This opinion, however, has not been favorably received. Dance, who has written one of the most complete and learned monographs on the formation of metastatic abscesses, believes that pus introduced into the circulation exerts a morbid action on the composition of the blood, which generates a predisposition to the formation of abscesses where there is any irritation.—Or in other words, it produces a pyogenic condition in the animal economy. Cruveilhier says that the pus globules are arrested in the capillaries, and thus become foci of irritation. This, with the alteration of the blood produced by pus, are interesting subjects ; but it would extend this article to too great length to enter further into their history. The preceding remarks might perhaps be regarded as irrelevant to our subject, or if not irrelevant, at least more applicable to ordinary phlebitis. We reply, that the nature and symptoms of puerperal suppurative phlebitis, with purulent infection, are identical with those of suppurative phlebitis from wounds and after surgical operations.

The internal surface of the uterus after parturition, presents all the appearances of a recent wound, especially that portion on which the placenta was attached. The veins open upon this surface, and are thus exposed to all the causes of inflammation and irritation incident to the parturient state.

The diagnosis of puerperal suppurative phlebitis presents few difficulties. This assertion may appear paradoxical, and therefore demands some explanation.

In the classification of puerperal diseases, which has already been adverted to, the opinion was hazarded that all the forms of puerperal disease have been and are now included under one head, viz.: Puerperal fever. And notwithstanding the vast learning which has been recently displayed in the debates in the French and the New York academies of medicine, on the nature of puerperal fever, nothing can, upon a logical analysis of these debates, be found to destroy the opinion maintained by Drs. Depaul and Simpson, that there is a puerperal fever essential in its nature, and which may run its career without any disorganization of structure. That a puerperal fever of this character is rare, we frankly admit; and we will go farther, and say that a lifetime may be passed in the study of diseases of females, without meeting with a case of this form of disease. It may be pronounced a great hardihood to assert that such physicians as Velpeau, Beare, Cayeau, Clark and Robert Lee have confounded, under one name, diseases which should be placed in a distinct nosological rank; and yet, if you will examine minutely the views of these distinguished men, you must come to the conclusion that puerperal fever consists in an inflammation of the peritoneum, or uterus, or veins, or all together. Similar confusion prevails in the various works on puerperal fever. Examine the treatises of Ashwell, Churchill, Meigs, and many others of equal authority, and the difficulty is forced upon the enquiring mind, not what puerperal fever is, but what it is not. This diversity of opinion would not be productive of much harm, if it did not affect the application of remedies. The theory of a particular disease is valuable, and to be appreciated alone for the knowledge it furnishes in the administration of remedies. Hence arises the great diversity of plans of treatment of puerperal fever. One proclaims that opium is our sheet anchor; another, that mercury is only to be relied on; and another, that the lancet is the "magnum donum dii."

A remark was once made to us by a physician of this city, now no more, who for grasp of intellect, profound erudition and practical acumen, would compare favorably with any of any age or country, that during an extensive practice of forty years, he never lost but one case of puerperal fever, and that was from culpable oversight. On enquiry what that oversight was, he with emphasis said, "I did not bleed her, sir." The truth was, that this eminent practitioner had been so fortunate as to meet with cases of inflammation of the peritoneum and uterus, of a high grade, in which the lancet was pre-eminently proper.

Again—There is a form of puerperal disease, which may be denominated "putrid infection," a very different disease from purulent infection. This disease arises from the reabsorption of putrid particles or principles. These putrid principles may exist in the air of crowded and ill-ventilated places, or they may emanate from the decomposition of organic matter. No doubt that the virulence and fatality of those epidemics so graphically described by Denman, Hulme, Kirkland and Ju[illegible]seu, were owing in part to the absorption of putrid principles, and their effects upon the composition of the blood.

The French medical school has not at this day shaken off the trammels of Broussaism. Local phlegmasiæ are too exclusively relied on in the interpretation of phenomena. In making this remark, let it not be a desire imputed to us directly or indirectly to depreciate morbid anatomy, for we acknowledge the great benefits which the cultivation of this department has conferred on practical medicine; but we do assert more errors are committed in the interpretation of autopsic phenomena than in any other branch of the science. We therefore concur in the opinion, so ably defended by Dr. Barker of the New York academy, that there is a puerperal fever without local phlegmasia, and that puerperal phlebitis, with or without purulent infection, is not puerperal fever.

In submitting the above remarks, it is not intended to exclude epidemic and atmospheric influences. But it may be urged, that if puerperal suppurative phlebitis, with purulent infection, be so formidable in its nature, why does it not present itself more frequently? The conditions which should produce it are found in almost every case of labor, and also, if the introduction of pus into the circulation produces such grave symptoms and such disastrous results, few lying-in women would escape. To these arguments, which have often been made by the ablest pathologists of this country, we reply, that there are two varieties of phlebitis—adhesive and suppurative phlebitis—and that adhesive phlebitis is a physiologico-pathological act, which prevents the introduction of pus by an obliteration of the veins; for, we concur with Raciborski, when he says, "that no woman recovers from accouchement, without adhesive phlebitis of the womb."

The experiments of Leuret, Halmont, and more recently, M Sedillot, are cited to show that pus can be injected into the veins without producing any serious symptoms; but any argument drawn from this source is clearly a *petitio principii.* These pathologists experimented upon animals in their healthy condition; and if their experiments prove any thing, they prove that pus affects the system not by its quantity, but by its quality; and whilst Dr. Sedillot could inject laudable pus with impunity, the smallest quantity of vitiated pus would give rise to symptoms of the greatest gravity. Moreover, we do not admit that this disease is so rare. There is no doubt in our mind that a large majority of sudden deaths in lying-in women would, upon an impartial examination, prove to be caused by suppurative phlebitis.—Inflammation of no organ or tissue produces death in twenty-four, thirty-six, or forty-eight hours.

It also must be borne in mind that suppurative phlebitis must occasion death without any autopsic revelations. Purulent deposits are not absolutely necessary to demonstrate the existence of pus in the blood, When, therefore, a woman is seized, one, two or three days after labor with a chill, followed by viscid perspiration, delirium (the circulating and respiratory organs being healthy, and with no symptoms of peritonitis or metritis), you may diagnose, with almost certainty, suppurative phlebitis.

With respect to the prognosis and treatment, but little can be said. The prognosis of puerperal suppurative phlebitis is awfully alarming; but few cases are on record where recovery has taken place. This is true relative to the disease after surgical operations. It is more so in lying-in women, where puerperal influence enters as an additional morbid element. We must acknowledge the impotency of our art in the treatment of this disease. To support the system and free the blood from poison, are the indications, to fulfill which, we must, in the language of the lamented Chapman, "exhaust the animal, vegetable and mineral kingdoms." But if we are impotent to cure, we are powerful to prevent. After the expulsion of the placenta, care should be taken to see that the womb has returned to its normal condition—in other words, that it is firmly contracted—as a firm, contracted womb favors adhesive phlebitis So long as the womb remains enlarged, engorged, or in a state of laxity, the woman will be exposed to phlebitis.

We remember reading in an English medical journal of the use of ergot, as a preventive of puerperal fever. We were struck with the article at the time, and it subsequently occurred to us, that if ergot could not prevent puerperal fever, it might prevent puerperal phlebitis.

It is a common practice for lying-in women to get up the ninth day, and with many, as was the case with our unfortunate patients, to get up on the 4th or 5th day. We have often witnessed bad effects from a too early rising after accouchement; and we are clearly of the opinion, that accidents would be less frequent, if patients would keep their beds for 15 or 20 days.

In conclusion—we would urge the adoption of every means to prevent the occurrence of this formidable malady. Secure a firm contraction of the womb by manipulations, bandages and ergot of rye; keep the patient confined to her bed for fifteen or twenty days; regulate the diet; keep the bowels open by mild laxatives; and combat, by energetic measures, the first appearance of disease.—*Vir. Medical Journal.*

